# Penicillin Disrupts Dental Mineralization in Rats: A Comparative Study With Tetracycline Highlighting Prenatal and Postnatal Risks

**DOI:** 10.1002/cre2.70225

**Published:** 2025-09-19

**Authors:** Sedigheh Mozafar, Fateme Mashhadi Abbas, Majid Mehran, Somayeh Kameli, Motahare Ahmadvand, Amir Mohammad Sharafi, Reza Omid, Morteza Banakar

**Affiliations:** ^1^ Department of Pediatric Dentistry, School of Dentistry Shahed University Tehran Iran; ^2^ Department of Oral and Maxillofacial Pathology, School of Dentistry Shahid Beheshti University of Medical Sciences Tehran Iran; ^3^ Dentist Private Dental Practice Tehran Iran; ^4^ Urology Research Center, Sina Hospital Tehran University of Medical Sciences Tehran Iran; ^5^ Department of Research Analytics, Saveetha Dental College and Hospitals, Saveetha Institute of Medical and Technical Sciences Saveetha University Chennai Tamil Nadu India; ^6^ Health Policy Research Center, Institute of Health Shiraz University of Medical Sciences Shiraz Iran

**Keywords:** amelogenesis, antibiotic exposure, dental development, enamel hypoplasia, molar‐incisor hypomineralization, rat model

## Abstract

**Objectives:**

Penicillin's impact on enamel defects remains understudied, particularly regarding its ability to cause structural issues even at therapeutic doses.

**Material and Methods:**

Pregnant Wistar rats received daily gavage from gestational day 13–22 with saline (control), 130 mg/kg tetracycline, 50 mg/kg penicillin, or 100 mg/kg penicillin. After birth, pups received the same treatment for 12 days. Upper first molars were analyzed for enamel/dentin thickness, maturation, and histopathological changes.

**Results:**

Tetracycline significantly reduced enamel and dentin thickness, disrupted their development, and caused flattening of the dentin‐enamel junction (DEJ) compared to the control group. Penicillin at 100 mg/kg also significantly decreased enamel and dentin thickness, impaired their maturation, and led to DEJ flattening. At 50 mg/kg, penicillin did not significantly affect enamel and dentin thickness but still disrupted their development and caused DEJ flattening.

**Conclusions:**

Penicillin at 100 mg/kg adversely affected enamel and dentin development, causing significant defects similar to those caused by tetracycline, although the severity and mechanisms may differ. Even at 50 mg/kg, penicillin disrupted enamel and dentin development, underscoring the clinical relevance of these findings and the need for caution when prescribing penicillin during pregnancy, even at lower doses, due to its potential to disrupt dental development.

## 1 Introduction

Ameloblasts play a crucial role in amelogenesis, the formation and maturation of enamel. These cells secrete the enamel matrix and contribute to the development of fully mineralized adult enamel (Pandya and Diekwisch 2021). Amelogenesis is tightly controlled but susceptible to environmental disturbances, such as fever, infection, trauma, hypoxia, and antibiotics. The long‐term consequences of these factors may manifest in enamel secretion and deposition, as the hard tooth structure cannot remodel (Bezamat et al. 2021). Enamel defects are categorized into two groups: (I) enamel hypoplasia, related to quantity, and (II) hypo‐mineralization, related to mineral content and degree of mineralization (Nowak et al. 2019; Seow 2014).

The term “Molar‐Incisor Hypo‐mineralization (MIH)” was first proposed in 2001 to describe developmental enamel abnormalities affecting one or more permanent molars, with or without the involvement of the incisors (Wuollet et al. 2016). In MIH, enamel maturation and calcification are hindered, leading to various abnormalities, including white‐yellow or yellow‐brown opacities and severely hypo‐mineralized enamel with a compromised structure (Lygidakis et al. 2022). Research indicates that antibiotic use during pregnancy significantly contributes to enamel abnormalities and the incidence of MIH (Acosta et al. 2022; Ardini et al. 2019; Elzein et al. 2021). Studies have also shown a link between antibiotics and MIH in Iran (Ahmadi et al. 2012; Khanmohammadi et al. 2022). Discussions have arisen about the potential for antibiotics to induce enamel abnormalities resembling fluorosis (Ravindra et al. 2023; Schmalfuss et al. 2022).

Dental hypo‐mineralization poses a challenge for dental professionals, arising from both environmental and genetic factors during enamel growth (Almulhim 2021). Existing literature on MIH primarily focuses on antibiotics, particularly amoxicillin and tetracycline. While some studies have not established a direct causative relationship between antibiotic use and MIH, they report a higher incidence of dental developmental defects associated with antibiotic treatment (Feltrin‐Souza et al. 2020; Kameli et al. 2019; Laisi et al. 2009).

Despite its perceived safety in pediatric infections, penicillin's effects on dental development remain poorly understood (Le Vavasseur and Zeller 2022; Lobanovska and Pilla 2017). Although its use is contraindicated for pregnant and lactating women, it is sometimes administered for serious infections, necessitating a thorough evaluation of its impact on dental development (Cantarutti et al. 2021; Schmalfuss et al. 2022).

To date, no research has investigated the association between penicillin and MIH (Brejawi et al. 2022; Juárez‐López et al. 2023). The prevalence of MIH varies globally, ranging from 5% to 25% in European countries (Damares Lago et al. 2022; Irigoyen‐Camacho et al. 2020; Lopes et al. 2021).

Given these factors, this investigation aims to evaluate the impact of prenatal and postnatal penicillin treatment, compared to tetracycline, on the composition and mineralization of enamel and dentin in rats.

## 2 Methods

### 2.1 Animal Housing and Breeding Protocol

Twenty female Wistar rats (age 2–3 months; weight 250 g) were used in this in vivo experimental study. All experimental procedures were conducted in accordance with the National Institutes of Health guidelines for the care and use of laboratory animals and were approved by the Ethics Committee of the School of Dentistry, Shahed University of Medical Sciences (Approval ID: IR.SHAHED.REC.1398.114). The experimental rats were maintained under controlled environmental conditions, with a temperature of 23 ± 1°C and humidity levels of 55% ± 5%. They were subjected to a light/dark cycle of 12 h each and were provided with unrestricted access to food and water. Subsequently, the male rats were paired with the female rats for mating purposes, and at an early hour in the morning, the male rats were separated from the enclosures. Samples of vaginal smears were obtained from the female rats and afterward examined using a light microscope. A veterinary reproductive specialist experienced in rodent breeding protocols, under the supervision of a pathologist, conducted the collection and examination of the smears to ensure accurate determination of conception. The assessment of sperm presence in the stained vaginal smears was conducted on the first day of conception (Umamageswari et al. 2020). Throughout the study, all efforts were made to minimize animal suffering. The rats were housed in a controlled environment with enrichment materials to reduce stress. Gavage was performed by trained personnel to ensure minimal discomfort. Animal health was monitored daily, and humane endpoints were established. At the end, the animals were euthanized using a method in accordance with the AVMA Guidelines on Euthanasia.

### 2.2 Experimental Groups and Treatment Protocols

**Figure 1:**
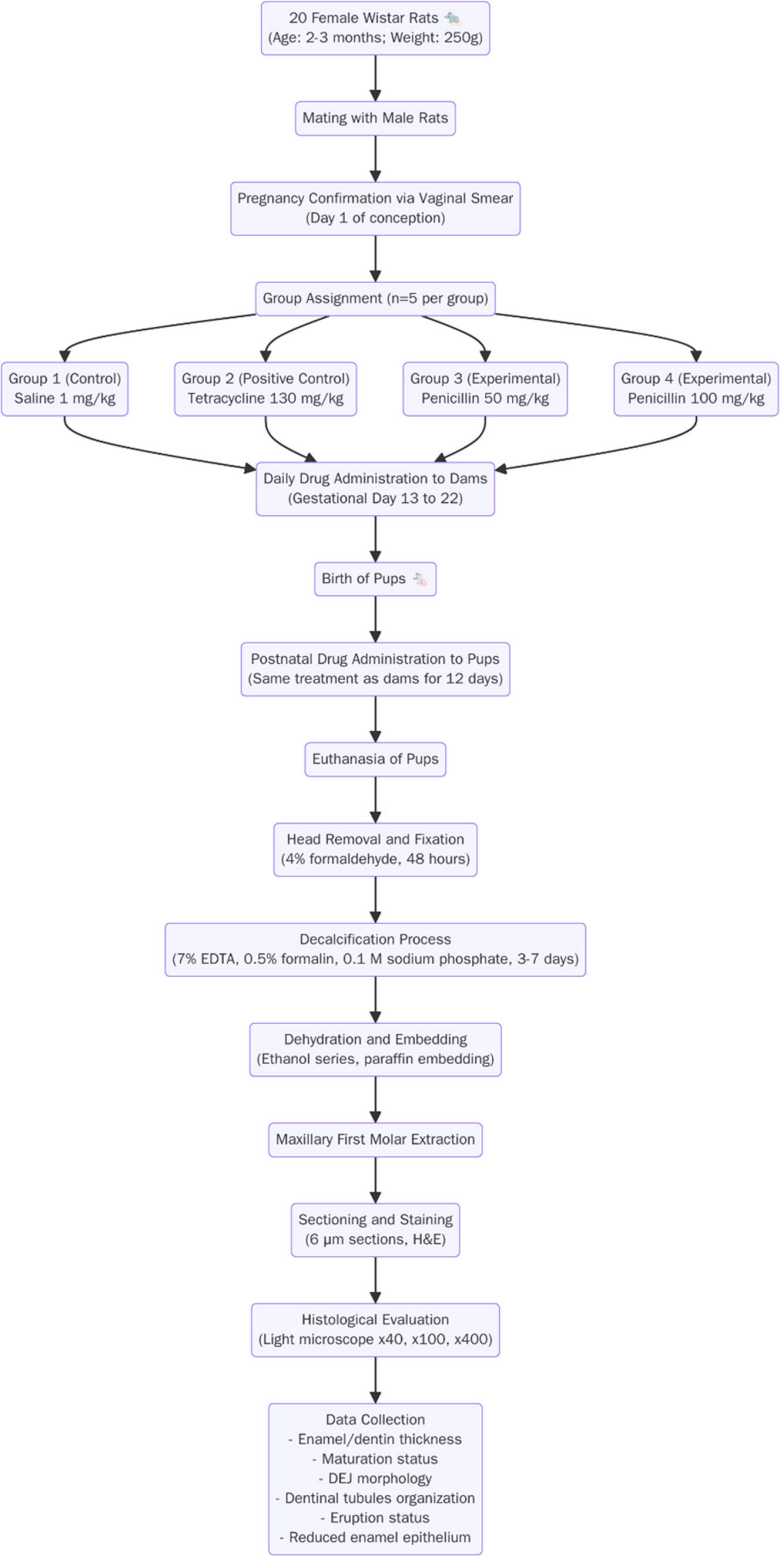
Illustrates the experimental design, including animal grouping, treatment protocols, and sample collection timeline. Pregnant Wistar rats were divided into four groups (*n* = 5 per group) and received daily gavage from gestational day 13–22. After birth, pups received the same treatment for 12 days before sample collection and analysis.

A total of 20 rats were randomly assigned to one of four groups (*n* = 5 per group). This sample size was determined by a power analysis (see Section 2.6). The groups were defined as follows (Figure 1).

#### 2.2.1 Negative Control Group

Received the vehicle (0.9% physiological saline) to control for procedural effects such as gavage and handling.

#### 2.2.2 Positive Control Group

Received 130 mg/kg of tetracycline. Tetracycline was selected as a classical reference for adverse effects on developing teeth. This dose was chosen as it is known to be toxic to developing teeth and reliably induces enamel hypoplasia and discoloration, serving as a benchmark for dental damage (Kim et al. 2022; Primosch 1980).

#### 2.2.3 Experimental Group 1

Received 50 mg/kg of penicillin.

#### 2.2.4 Experimental Group 2

Received 100 mg/kg of penicillin.

The selection of antibiotic dosages was based on establishing clinically relevant and supratherapeutic exposure levels to assess a dose–response relationship. The penicillin doses were chosen to model distinct clinical scenarios. The 50 mg/kg dose represents a high‐end therapeutic exposure, comparable to the upper limits of the typical human therapeutic range of 25–50 mg/kg/day (Brunton et al. 2018). Using allometric scaling (Reagan‐Shaw et al. 2008), the human‐equivalent dose (HED) for 50 mg/kg in rats is ~8 mg/kg, aligning with the lower human therapeutic range. Conversely, the 100 mg/kg dose (HED: ~16 mg/kg) represents a supratherapeutic level, modeling accidental overdose or maximal toxicity.

The use of antibiotic doses in rats that are significantly higher than human mg/kg equivalents is a standard and necessary methodological approach in preclinical pharmacology. Rodents have a substantially higher metabolic rate than humans, leading to much faster drug clearance. To achieve systemic drug exposures (*AUC*) and resulting biological effects in rats that are comparable to those from therapeutic doses in humans, higher weight‐adjusted doses are required (Reagan‐Shaw et al. 2008). This principle of allometric scaling justifies the elevated doses used in this study, ensuring that the findings are relevant despite the physiological differences between species. This approach is well‐documented in preclinical studies investigating antibiotic‐induced toxicity or microbiome shifts (Lankelma et al. 2017). Figure 1 summarizes the experimental design and sampling process, including animal grouping, drug administration schedule, and histological evaluation steps.

### 2.3 Drug Administration Schedule

The rats were administered the designated drugs through gavage daily, starting from day 13 and continuing until day 22 of pregnancy. Following parturition, the neonatal rats were administered an identical pharmaceutical treatment as that given to their respective maternal subjects for a duration of 12 days. Treatment began on gestational day 13 to coincide with maxillary first molar development and ensure placental penicillin transfer (Schmalfuss et al. 2022). Furthermore, the present period marks the beginning of the growth process of the maxillary first molars in rats. The first molars in 12‐day‐old rats reach the last secretory stage in the cervical region and begin the process of enamel mineralization in the cusps. Furthermore, it has been shown that the active eruption process of the first molars starts at 12 days, and this phase is distinguished by the occurrence of mucosal perforation (Kameli et al. 2019).

### 2.4 Sample Processing

The rats from all experimental groups were euthanized after a 12‐day period which is sufficient to capture early‐stage enamel defects (Abe et al. 2003). Rats were euthanized via carbon dioxide asphyxiation followed by cervical dislocation, a method approved by the ethics committee to ensure minimal suffering. Subsequently, their heads were removed and placed in a solution of 4% formaldehyde at room temperature for 48 h to achieve fixation. Following the process of decalcification in a solution containing 7% disodium ethylene diamine tetra‐acetic acid, 0.5% formalin, and 0.1 M sodium phosphate over a period of 3–7 days, the samples were subjected to dehydration using a series of ethanol solutions with varying percentages. Finally, the samples were embedded in paraffin (de Souza et al. 2016). Then, the molars underwent a surgical extraction procedure and were submerged in a formaldehyde solution.

### 2.5 Examination of Enamel Formation

The maxillary first molars were sliced into sections that were 6 µm thick using a low‐speed micro‐motor. The slicing was performed in the frontal plane, parallel to the axial plane of the teeth. During sectioning, precise angulation adjustments were made, followed by staining the sections with hematoxylin and eosin. The specimens were then examined by a pathologist using a light microscope (Reichert Jung DiaStar; Cambridge Instruments, Buffalo, NY, USA) at magnifications of ×40, ×100, and ×400. The morphological assessment of the ameloblasts, which are structured in a single layer of cylindrical cells, was conducted (de Souza et al. 2016). The pathologist, who had been calibrated through standardized training sessions to ensure consistency in histological assessments, was blinded to the group allocation of the material. A total of three sections per molar were analyzed to derive the reported measurements. The assessment included recording various histological and clinical alterations, such as dentin and enamel thickness, dentin and enamel maturation, eruption status, presence of reduced enamel epithelium (REE), DEJ status, and dentinal tubules. Enamel and dentin were classified as “mature” when showing uniform mineral density, well‐organized prism or tubule structure, and absence of interglobular areas; and as “immature” when exhibiting incomplete mineralization, irregular prism/tubule orientation, vacuolization or hypomineralization (indicated by altered staining) (Farci and Soni 2021; Wada et al. 2023). These criteria were also adapted from de Souza et al. (2016) and Kameli et al. (2019) (de Souza et al. 2016; Kameli et al. 2019).

### 2.6 Statistical Analysis

The data underwent analysis with SPSS version 22. The sample size of 5 animals per group (*n* = 20 total) was calculated based on effect size estimates from previous enamel toxicology studies (de Souza et al. 2016; Kameli et al. 2019), aiming for a power of 80% and *α* = 0.05 to detect a 20% change in enamel thickness. The Shapiro–Wilk test was used to assess the normality of the data distribution. The data were presented in terms of frequency and percentage, and were subjected to statistical analysis using the Chi‐square test and the Kruskal‐Wallis test to compare the groups.

## 3 Results

### 3.1 Histological Findings

A total of ten specimens were assessed within each group. In the sample of 40 specimens, it was observed that 50% (*n* = 20) exhibited mature enamel and dentin, whereas the remaining 50% (*n* = 20) had immature enamel and dentin. The eruption status of the specimens was assessed based on a sample size of 40. Out of them, 24 specimens (60%) exhibited no eruption, while 10 specimens (25%) displayed partial eruption, and six specimens (15%) showed full eruption. Regarding the condition of the reticular epithelium (REE), it was observed that out of a total of 40 specimens, the formation of the reticular epithelium was complete in 36 specimens (90%), whereas in four specimens (10%), the formation had not yet occurred. The dentinal tubules exhibited a consistent and organized arrangement across all specimens. The DEJ was scalloped in 15 specimens, accounting for 37.5% of the total. In 10 specimens, the DEJ was found to be flat, representing 25% of the total. Additionally, a combination of scalloped and flat DEJ was observed in 15 specimens, also accounting for 37.5% of the total. The average thickness of enamel across the various groups is presented in Table 1.

**Table 1:** Enamel and dentin thickness (mm) across groups (*n* = 10).

Tissue type	Experimental group	Mean thickness (mm)	SD	Minimum (mm)	Maximum (mm)
**Enamel**	Penicillin 100 mg/kg	0.112	0.035	0	1.12
	Penicillin 50 mg/kg	0.114	0.006	0.1	0.12
	Tetracycline 130 mg/kg	0.065	0.025	0.04	0.11
	Saline	0.119	0.009	0.10	0.13
**Dentin**	Penicillin 100 mg/kg	0.076	0.022	0.04	0.10
	Penicillin 50 mg/kg	0.123	0.006	0.11	0.13
	Tetracycline 130 mg/kg	0.079	0.025	0.05	0.12
	Saline	0.101	0.005	0.09	0.11

*Note:* Values are presented as mean ± SD. Significant intergroup differences were observed for both enamel and dentin thickness (Kruskal–Wallis, *p* < 0.001).

### 3.2 Histopathological and Quantitative Analysis

**Figure 2:**
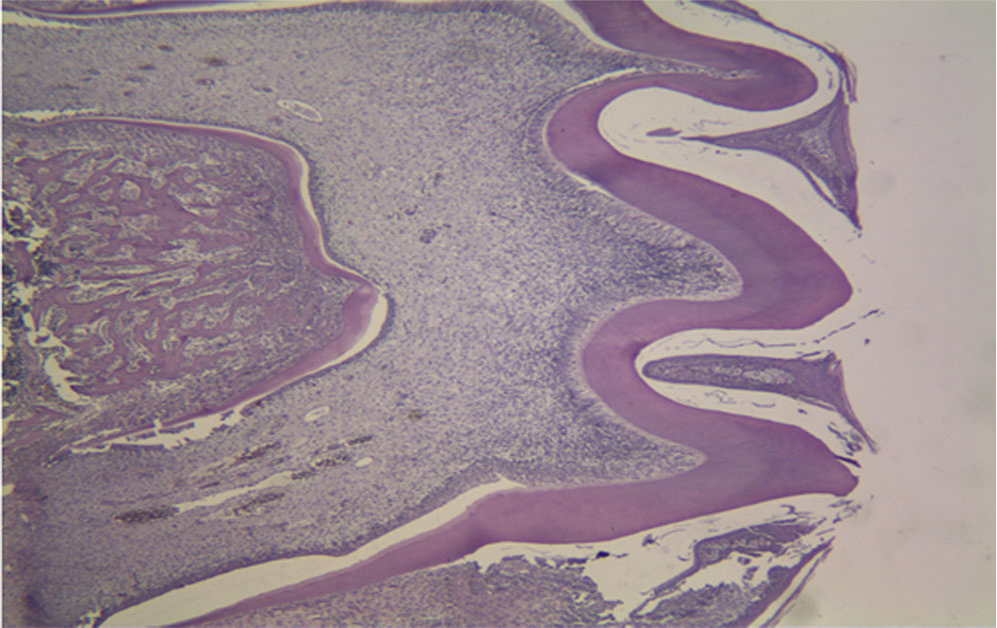
Histopathological examination of maxillary first molars from the saline control group in Wistar rats. Representative micrograph (40× magnification) showing normal dental tissue architecture following prenatal (gestational days 13–22) and postnatal (12 days) administration of physiological saline (0.9%) via oral gavage. The specimen demonstrates regular scalloping at the dentin–enamel junction (DEJ), uniform enamel thickness, mature enamel with complete mineralization, well‐organized enamel prisms, and normal ameloblast morphology. Dentin shows regular tubular structure with no evidence of hypomineralization. The reduced enamel epithelium (REE) is intact, and the dentinal tubules exhibit consistent organization throughout the dentin layer. This image represents the expected normal histological appearance of developing rat molars without antibiotic exposure, serving as the baseline for comparison with experimental groups.

**Figure 3:**
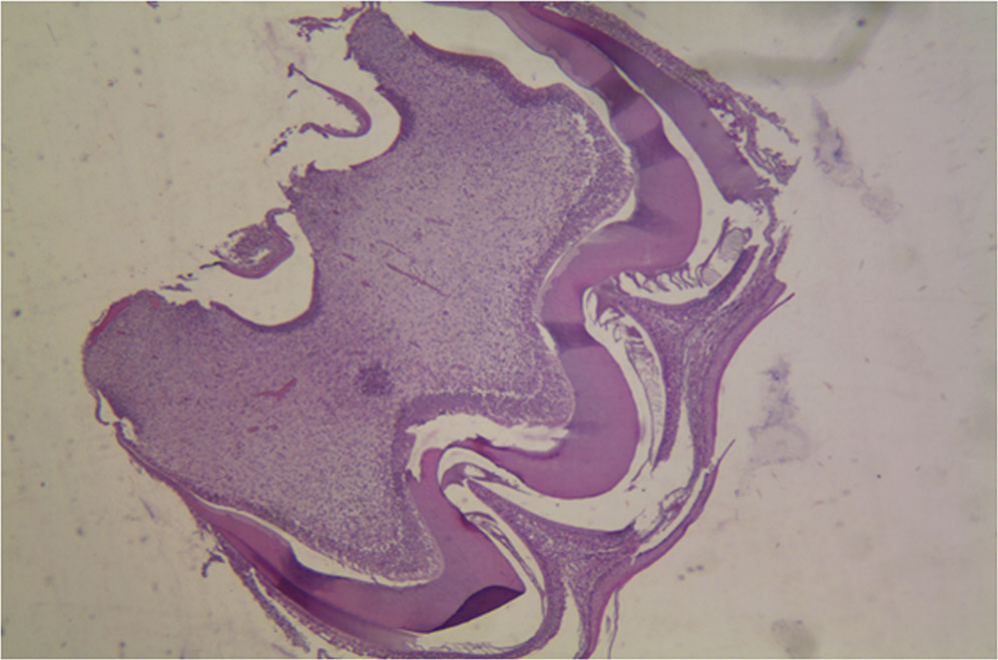
Histopathological examination of maxillary first molars from the 50 mg/kg penicillin group in Wistar rats. Representative micrograph (40× magnification) showing dental tissue architecture following prenatal (gestational days 13–22) and postnatal (12 days) administration of penicillin at 50 mg/kg via oral gavage. While enamel thickness shows no significant reduction compared to controls, the specimen demonstrates flattened dentin‐enamel junction (DEJ), subtle irregularities in enamel prism organization, and mild alterations in ameloblast morphology compared to the saline control group. The enamel shows areas of incomplete mineralization indicated by altered staining intensity, suggesting early hypo‐mineralization despite preserved tissue thickness. Dentin thickness is slightly increased, possibly reflecting compensatory mechanisms. This image demonstrates subclinical dental developmental changes induced by penicillin at therapeutic‐equivalent doses that may not be apparent through gross morphological assessment alone.

**Figure 4:**
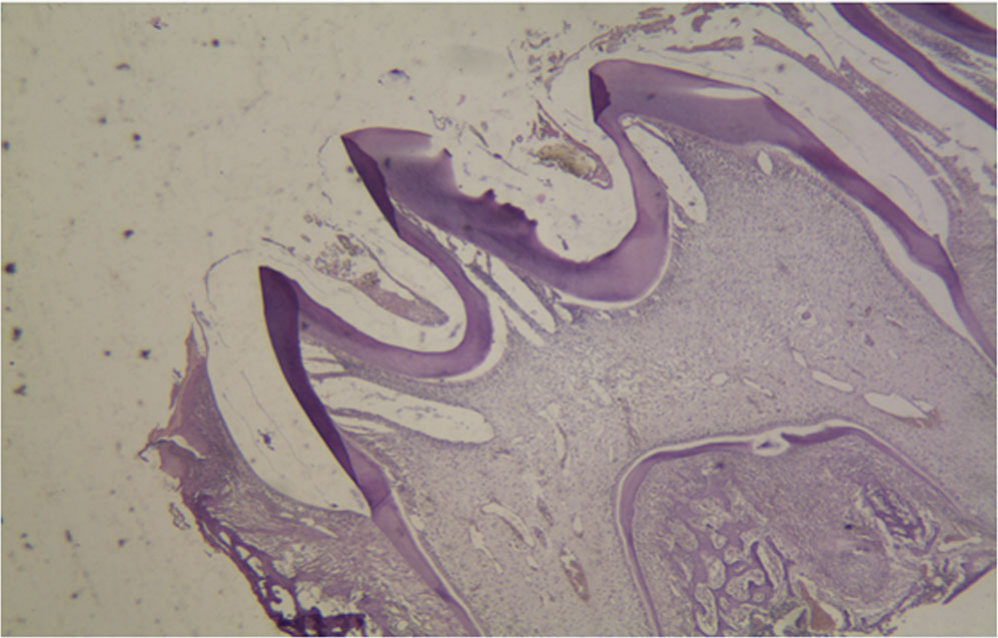
Histopathological examination of maxillary first molars from the 100 mg/kg penicillin group in Wistar rats. Representative micrograph (40× magnification) showing significant dental tissue alterations following prenatal (gestational days 13–22) and postnatal (12 days) administration of penicillin at 100 mg/kg via oral gavage. The specimen demonstrates markedly reduced enamel thickness with areas of hypoplasia, pronounced flattening of the dentin‐enamel junction (DEJ), and extensive areas of enamel hypo‐mineralization indicated by irregular staining patterns. Ameloblast morphology shows significant disruption with vacuolization and irregular arrangement. Dentin thickness is reduced with evidence of disrupted tubular organization in some regions. This image illustrates the dose‐dependent effects of penicillin on dental development, demonstrating that supratherapeutic doses cause structural and mineralization defects comparable to, though potentially mechanistically distinct from, tetracycline‐induced damage.

**Figure 5:**
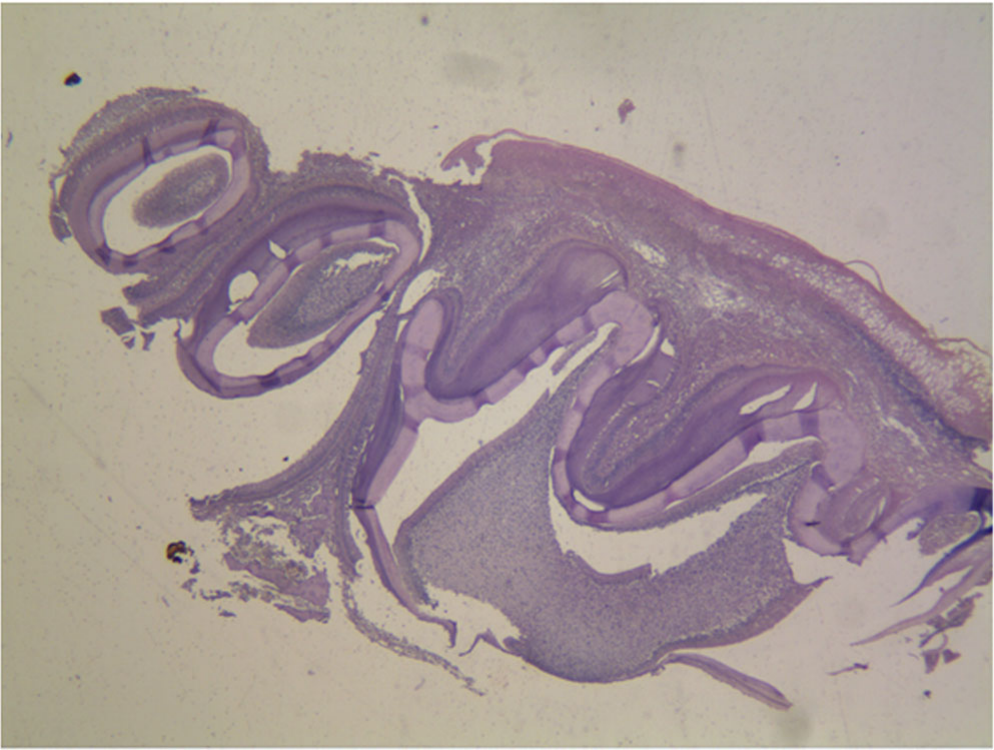
Histopathological examination of maxillary first molars from the 130 mg/kg tetracycline group in Wistar rats. Representative micrograph (40× magnification) showing severe dental tissue alterations following prenatal (gestational days 13–22) and postnatal (12 days) administration of tetracycline at 130 mg/kg via oral gavage. The specimen demonstrates significantly reduced enamel thickness with pronounced hypoplasia, complete loss of the normal scalloped dentin‐enamel junction (DEJ) morphology, and extensive enamel hypo‐mineralization. Ameloblasts show severe morphological disruption with evidence of apoptosis. Dentin thickness is reduced with an irregular tubular structure. Scale bar = 100 μm. This image represents the classic tetracycline‐induced dental defects used as a positive control in this study, demonstrating the well‐documented adverse effects of tetracycline on developing dental tissues through direct binding to calcium in mineralizing structures. The severity of these changes provides a benchmark for evaluating the comparative effects of penicillin on dental development.

The histopathological micrographs provide key insights into the effects of various treatments on dental structures. Figure 2 displays the saline group at x40 magnification, showing normal enamel and dentin structure with healthy ameloblast morphology. Figure 3 illustrates the 50 mg/kg penicillin group, revealing minor alterations in enamel thickness and slight changes in ameloblast arrangement. Figure 4 depicts the 100 mg/kg penicillin group, demonstrating more pronounced changes in ameloblast morphology and increased enamel irregularities. Finally, Figure 5 shows the 130 mg/kg tetracycline group, highlighting significant enamel damage, reduced dentin quality, and extensive alterations in the DEJ.

**Figure 6:**
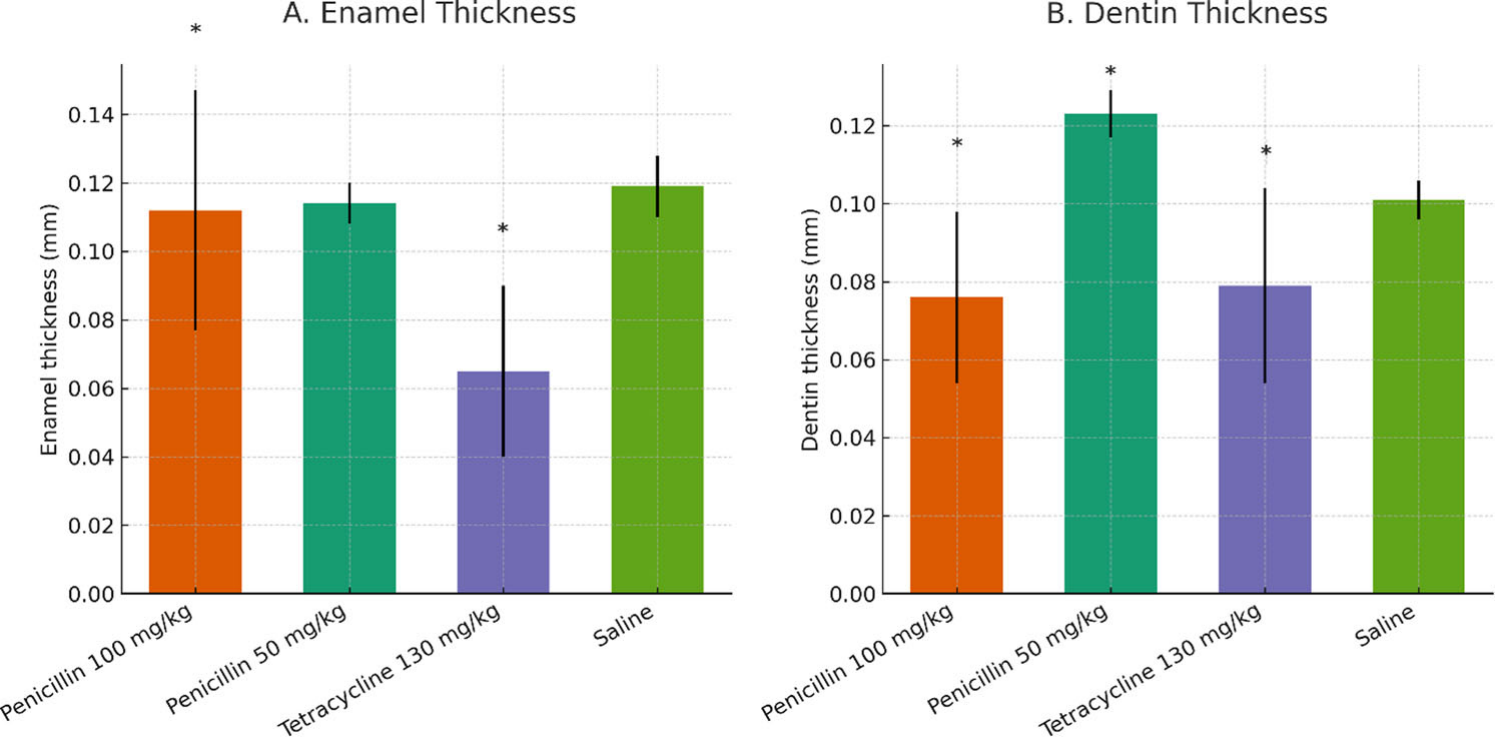
Bar plots of (A) enamel and (B) dentin thickness (mm) across experimental groups. Data are mean ± SD (*n* = 10 per group). Asterisks (*) indicate statistically significant differences (*p* < 0.05, Mann–Whitney U test) compared to the saline control or between specified groups, based on pairwise comparisons. Penicillin at 100 mg/kg and tetracycline significantly reduced enamel thickness compared to saline, while penicillin at 50 mg/kg showed no significant change. For dentin thickness, both penicillin doses and tetracycline showed significant differences from saline, with additional differences observed between penicillin doses.

The highest enamel thickness was seen in the group treated with saline, whereas the smallest enamel thickness was observed in the group treated with tetracycline. Table 2 presents the average dentin thickness values for the various groups. Mean enamel and dentin thickness values across groups are shown in Figure 6. The results indicate that the dentin thickness was highest in the group administered 50 mg/kg of penicillin, whereas the lowest dentin thickness was seen in the tetracycline group. Histopathological pictures of the research groups are shown in Figures 2, 3, 4, 5. The Shapiro–Wilk test indicated non‐normal distribution for enamel thickness in the tetracycline group (*p* = 0.008), dentin thickness in the saline group (*p* = 0.004), and others (*p* < 0.05). This justified the use of non‐parametric tests (Kruskal–Wallis, Mann–Whitney) for group comparisons.

**Table 2:** Pairwise comparisons of the groups were performed using the Mann‐Whitney U test regarding the Enamel thickness (*p* < 0.05 = significant).

Group 1	Group 2	Mean difference	Mann–Whitney U	*p* value
Saline	Penicillin 50 mg/kg	0.005	33.5	0.184
	Penicillin 100 mg/kg	0.007	21.0	0.001
	Tetracycline 130 mg/kg	0.054	2.500	< 0.001
Penicillin 50 mg/kg	Penicillin 100 mg/kg	0.002	10	0.001
	Tetracycline 130 mg/kg	0.049	3.500	< 0.001
Penicillin 100 mg/kg	Tetracycline 130 mg/kg	0.047	10	0.001

The results of the Kruskal‐Wallis test indicated a statistically significant disparity in both enamel (*p* < 0.001) and dentin thickness (*p* < 0.001) among the various groups. The Mann–Whitney test was used to conduct pairwise comparisons between the groups concerning enamel thickness (Table 2) and dentin thickness (Table 3). Significant variations in enamel thickness were seen across all groups (*p* < 0.05), except for the 50 mg/kg penicillin and saline groups (*p* > 0.05). Significant variations in dentin thickness were seen across all groups (*p* < 0.05), except for the groups administered 100 mg/kg penicillin and 130 mg/kg tetracycline (*p* > 0.05).

**Table 3:** Pairwise comparisons of the groups were performed using the Mann–Whitney U test regarding the Dentin thickness (*p* < 0.05 = significant).

Group I	Group 2	Mean difference	Mann–Whitney U	*p* value
Saline	Penicillin 50 mg/kg	−0.022	1.0	< 0.001
	Penicillin 100 mg/kg	0.025	12.0	0.001
	Tetracycline 130 mg/kg	0.022	24.50	0.046
Penicillin 50 mg/kg	Penicillin 100 mg/kg	0.047	0.00	< 0.001
	Tetracycline 130 mg/kg	0.044	7.0	0.001
Penicillin 100 mg/kg	Tetracycline 130 mg/kg	−0.003	48.50	0.909

## 4 Discussion

Antibiotic use during pregnancy may damage enamel development and result in molar‐incisor hypo‐mineralization (MIH) formation (Acosta et al. 2022). This study examined the effects of penicillin in high and low concentrations on enamel and dentin maturation compared to saline. Antibiotics impact enamel maturation through various mechanisms, primarily by suppressing protein synthesis. For instance, amoxicillin has been shown to lower metalloproteinase‐20 production, which is crucial for enamel maturation (Acosta et al. 2022).

Additionally, antibiotics can inhibit enamel secretion by ameloblasts, leading to vacuole‐like structures that impede protein production and severely disrupt enamel maturation (Feltrin‐Souza et al. 2020; Yoshizaki et al. 2020).

Tetracycline disrupts matrix protein release via its acidic properties, causing post‐mature enamel defects such as hypoplasia and amelogenesis imperfecta (Omnell et al. 1970; Sasaki et al. 1990). The current findings indicate that tetracycline altered the characteristic scalloping pattern of the DEJ, with flat structures observed in 20% of cases. This aligns with previous research, confirming tetracycline's impact on DEJ morphology (Bjorvatn 1983; Radlanski and Renz 2007).

Regarding penicillin, the administration of 100 mg/kg significantly disrupted enamel maturation, leading to thinner enamel and alterations in dentin characteristics, including flat DEJ structures in 40% of instances. Conversely, the 50 mg/kg penicillin dose, while not significantly altering tissue thickness, still induced developmental disruptions, such as replacing the normal scalloped DEJ with flat structures. These subclinical changes suggest that even lower therapeutic doses may act as precursors to MIH, underscoring the dose‐dependent effects of penicillin. The findings emphasize the dose‐dependent effects of penicillin, indicating that a lesser influence on ameloblast differentiation may explain the reduced effects at 50 mg/kg. This raises critical clinical implications regarding the use of penicillin during pregnancy. Given the risk of permanent enamel/dentin defects alternatives (e.g., erythromycin) should be considered.

Although 50 mg/kg penicillin did not reduce enamel/dentin thickness, histopathological changes suggest subclinical defects, underscoring the need for microscopic evaluation. These changes can precede or occur independently of gross thickness reduction; microscopic alterations such as hypo‐mineralization, vacuolization of odontoblastic or ameloblastic layers, and disruptions in cellular differentiation can compromise tissue quality and function without immediately translating to measurable thickness loss (Gottberg et al. 2014; Sabah and Al‐Ghaban 2023). Studies have shown that antibiotics like amoxicillin can disrupt mineralization and cellular integrity even when tissue volume remains unchanged (Feltrin‐Souza et al. 2020; Kameli et al. 2019). This suggests that penicillins impact the quality of enamel and dentin through mechanisms such as altered protein synthesis and impaired cell differentiation. Additionally, lower or shorter exposures may not cause gross hypoplasia but can still disrupt the microarchitecture of developing dental tissues, leading to functional defects detectable only histologically (Feltrin‐Souza et al. 2020; Gottberg et al. 2014; Kameli et al. 2019). Clinically, these histopathological changes can predispose teeth to increased wear, caries, or esthetic problems, emphasizing that relying solely on thickness measurements may underestimate the true impact of drug exposure on dental development (Cossa et al. 2020; Shekarchizadeh et al. 2013). In summary, this finding highlights that penicillin can cause significant subclinical, or microscopic dental defects without overtly reducing enamel or dentin thickness, underscoring the need for comprehensive histological evaluation when assessing drug safety during tooth development.

This study's strengths include the comparison of tetracycline and two different doses of penicillin on dental growth, using saline as a control. However, limitations exist, including the challenge of translating findings from rat models to humans, given differences in dental development timelines and antibiotic metabolism. Additionally, the lack of molecular analyses, such as matrix protein expression, limits our understanding of the mechanisms involved. Furthermore, the differences in enamel formation timelines between rats and humans present significant translational challenges, as the developmental processes and responses to antibiotics may not directly translate to clinical implications for human dental health. Future research should investigate the molecular pathways affected by penicillin, particularly the expression of metalloproteinase‐20, and evaluate its effects in human cohort studies. Understanding these mechanisms will be crucial for developing guidelines for antibiotic use during pregnancy to minimize risks to dental health in offspring.

## 5 Conclusion

This study demonstrates that prenatal and postnatal exposure to penicillin, particularly at a supratherapeutic dose (100 mg/kg), induces significant enamel and dentin mineralization defects in rats, compared to the adverse effects observed with tetracycline. Histopathological analysis revealed dose‐dependent disruptions in dental tissue maturation, including flattened dentin‐enamel junctions (DEJ) and reduced enamel/dentin thickness at higher doses. Notably, even the lower penicillin dose (50 mg/kg) caused subclinical structural abnormalities, underscoring its potential to compromise dental development despite minimal changes in tissue thickness. These findings highlight the clinical relevance of penicillin's adverse effects on amelogenesis and dentinogenesis, emphasizing the need for caution when prescribing this antibiotic during pregnancy or early childhood, even at therapeutic doses.

## Author Contributions

S.M. and A.M.S. contributed to the study conception and design, analyzed data, and edited the manuscript. M.B., R.O., and F.M.A. contributed to the study design and wrote the first draft of the manuscript. M.M., M.A., S.K., and S.M. contributed to the study design, data collection, and drew figures. All authors commented on previous versions of the manuscript and revised it. All authors read and approved the final manuscript.

## Ethics Statement

The study was carried out following the guidelines for the care and use of laboratory animals. The research received approval from the ethics committee of the School of Dentistry at Shahed University of Medical Sciences. (Approval ID: IR.SHAHED. REC.1398.114).

## Conflicts of Interest

The authors declare no conflicts of interest.

## Data Availability

The datasets used and/or analyzed during the current study are available from the corresponding author upon reasonable request.

## References

[cre270225-bib-0001] Abe, T. , H. Miyajima , and K. Okada . 2003. “Effects of a Macrolide Antibiotic on Enamel Formation in Rat Incisors‐Primary Lesion of Ameloblast at the Transition Stage.” Journal of Veterinary Medical Science 65, no. 9: 985–988. 14532690 10.1292/jvms.65.985

[cre270225-bib-0002] Acosta, E. , O. Cortes , S. Guzman , M. Catala , M. Lorente , and J. J. Arense . 2022. “Relationship Between Molar Incisor Hypomineralization, Intrapartium Medication and Illnesses in the First Year of Life.” Scientific Reports 12, no. 1: 1637. 10.1038/s41598-022-05628-7. 35102194 PMC8803910

[cre270225-bib-0003] Ahmadi, R. , N. Ramazani , and R. Nourinasab . 2012. “Molar Incisor Hypomineralization: A Study of Prevalence and Etiology in a Group of Iranian Children.” Iranian Journal of Pediatrics 22, no. 2: 245–251. 23056894 PMC3446062

[cre270225-bib-0004] Almulhim, B. 2021. “Molar and Incisor Hypomineralization.” Journal of the Nepal Medical Association 59, no. 235: 295–302. 10.31729/jnma.6343. 34506432 PMC8369532

[cre270225-bib-0005] Ardini, Y. D. , N. N. Ismail , N. D. M. Azni , and N. A. Harun . 2019. “Molar Incisor Hypomineralisation: Prevalence and Associated Risk Factors Among Children at the Polyclinic, Kulliyyah of Dentistry, Iium.” Materials Today: Proceedings 16: 2351–2356. 10.1016/j.matpr.2019.06.138.

[cre270225-bib-0006] Bezamat, M. , J. F. Souza , F. M. F. Silva , et al. 2021. “Gene‐Environment Interaction in Molar‐Incisor Hypomineralization.” PLoS One 16, no. 1: e0241898. 10.1371/journal.pone.0241898. 33406080 PMC7787379

[cre270225-bib-0007] Bjorvatn, K. 1983. “In Vitro Study by Fluorescence Microscopy and Microradiography of Tetracycline‐Tooth Interaction.” Scandinavian Journal of Dental Research 91, no. 6: 417–424. 10.1111/j.1600-0722.1983.tb00840.x. 6581516

[cre270225-bib-0008] Brejawi, M. S. , A. Venkiteswaran , S. M. O. Ergieg , and B. M. Sabri . 2022. “Correlation Between Molar‐Incisor Hypomineralization, Stress, and Family Functioning.” Journal of International Society of Preventive and Community Dentistry 12, no. 5: 547–553. 10.4103/jispcd.JISPCD_105_22. 36532319 PMC9753921

[cre270225-bib-0009] Brunton, L. L. , B. C. Knollmann , and R. Hilal‐Dandan . 2018. Goodman & Gilman's the Pharmacological Basis of Therapeutics (13). McGraw‐Hill Education.

[cre270225-bib-0010] Cantarutti, A. , F. Rea , M. Franchi , B. Beccalli , A. Locatelli , and G. Corrao . 2021. “Use of Antibiotic Treatment in Pregnancy and the Risk of Several Neonatal Outcomes: A Population‐Based Study.” International Journal of Environmental Research and Public Health 18, no. 23: 12621. 10.3390/ijerph182312621. 34886350 PMC8657211

[cre270225-bib-0011] Cossa, F. , A. Piastra , M. Sarrion‐Pérez , and L. Bagán . 2020. “Oral Manifestations in Drug Users: A Review.” Journal of Clinical and Experimental Dentistry 12, no. 2: e193–e200. 10.4317/jced.55928. 32071702 PMC7018485

[cre270225-bib-0012] Damares Lago, J. , M. Restrepo , D. Girotto Bussaneli , et al. 2022. “Molar‐Incisor Hypomineralization: Prevalence Comparative Study in 6 Years of Interval.” Scientific World Journal 2022: 4743252. 10.1155/2022/4743252. 36530554 PMC9757933

[cre270225-bib-0013] Elzein, R. , E. Chouery , F. Abdel‐Sater , R. Bacho , and F. Ayoub . 2021. “Molar‐Incisor Hypomineralisation in Lebanon: Association With Prenatal, Natal and Postnatal Factors.” European Archives of Paediatric Dentistry 22, no. 2: 283–290. 10.1007/s40368-020-00555-5. 32889651

[cre270225-bib-0014] Farci, F. , and A. Soni . 2021. Histology, Tooth. 34283421

[cre270225-bib-0015] Feltrin‐Souza, J. , F. Jeremias , S. Alaluusua , et al. 2020. “The Effect of Amoxicillin on Dental Enamel Development In Vivo.” Brazilian Oral Research 34: e116. 10.1590/1807-3107bor-2020.vol34.0116. 32901731

[cre270225-bib-0016] Gottberg, B. , J. Berne , B. Quinonez , and E. Solorzano . 2014. “Prenatal Effects by Exposing to Amoxicillin on Dental Enamel in Wistar Rats.” Medicina Oral Patología Oral y Cirugia Bucal 19, no. 1: e38–e43. 10.4317/medoral.18807. 24121904 PMC3909430

[cre270225-bib-0017] Irigoyen‐Camacho, M. E. , T. Villanueva‐Gutierrez , A. Castano‐Seiquer , N. Molina‐Frechero , M. Zepeda‐Zepeda , and L. Sánchez‐Pérez . 2020. “Evaluating the Changes in Molar Incisor Hypomineralization Prevalence: A Comparison of Two Cross‐Sectional Studies in Two Elementary Schools in Mexico City Between 2008 and 2017.” Clinical and Experimental Dental Research 6, no. 1: 82–89. 10.1002/cre2.252. 32067391 PMC7025996

[cre270225-bib-0018] Juárez‐López, M. L. A. , L. V. Salazar‐Treto , B. Hernández‐Monjaraz , and N. Molina‐Frechero . 2023. “Etiological Factors of Molar Incisor Hypomineralization: A Systematic Review and Meta‐Analysis.” Dentistry Journal 11, no. 5: 111. 37232762 10.3390/dj11050111 PMC10217283

[cre270225-bib-0019] Kameli, S. , N. Moradi‐Kor , R. Tafaroji , R. Ghorbani , H. Farzadmnesh , and H. Sameni . 2019. “Effects of Amoxicillin on the Structure and Mineralization of Dental Enamel and Dentin in Wistar Rats.” Frontiers in Dentistry 16, no. 2: 130–135. 10.18502/fid.v16i2.1364. 31777854 PMC6874840

[cre270225-bib-0020] Khanmohammadi, R. , B. Seraj , A. Salari , and F. Alipour . 2022. “Etiological Factors Involved in Molar‐Incisor Hypomineralization in 7 to 12‐Year‐Old Children in Tehran.” Frontiers in Dentistry 19: 16. 10.18502/fid.v19i16.9962. 36458266 PMC9675622

[cre270225-bib-0021] Kim, S. J. , E. H. Kim , M. Lee , et al. 2022. “Risk of Dental Discoloration and Enamel Dysplasia in Children Exposed to Tetracycline and Its Derivatives.” Yonsei Medical Journal 63, no. 12: 1113–1120. 10.3349/ymj.2022.0388. 36444547 PMC9760895

[cre270225-bib-0022] Laisi, S. , A. Ess , C. Sahlberg , P. Arvio , P. L. Lukinmaa , and S. Alaluusua . 2009. “Amoxicillin May Cause Molar Incisor Hypomineralization.” Journal of Dental Research 88, no. 2: 132–136. 10.1177/0022034508328334. 19278983

[cre270225-bib-0023] Lankelma, J. M. , L. A. van Vught , C. Belzer , et al. 2017. “Critically Ill Patients Demonstrate Large Interpersonal Variation in Intestinal Microbiota Dysregulation: A Pilot Study.” Intensive Care Medicine 43, no. 1: 59–68. 27837233 10.1007/s00134-016-4613-z PMC5203863

[cre270225-bib-0024] Lobanovska, M. , and G. Pilla . 2017. “Penicillin's Discovery and Antibiotic Resistance: Lessons for the Future?” Yale Journal of Biology and Medicine 90, no. 1: 135–145. 28356901 PMC5369031

[cre270225-bib-0025] Lopes, L. B. , V. Machado , P. Mascarenhas , J. J. Mendes , and J. Botelho . 2021. “The Prevalence of Molar‐Incisor Hypomineralization: A Systematic Review and Meta‐Analysis.” Scientific Reports 11, no. 1: 22405. 10.1038/s41598-021-01541-7. 34789780 PMC8599453

[cre270225-bib-0026] Lygidakis, N. A. , E. Garot , C. Somani , G. D. Taylor , P. Rouas , and F. S. L. Wong . 2022. “Best Clinical Practice Guidance for Clinicians Dealing With Children Presenting With Molar‐Incisor‐Hypomineralisation (MIH): An Updated European Academy of Paediatric Dentistry Policy Document.” European Archives of Paediatric Dentistry 23, no. 1: 3–21. 10.1007/s40368-021-00668-5. 34669177 PMC8926988

[cre270225-bib-0027] Nowak, A. J. , J. R. Christensen , T. R. Mabry , J. A. Townsend , and M. H. Wells . 2019. Pediatric Dentistry: Infancy Through Adolescence (10). Elsevier.

[cre270225-bib-0028] Omnell, K. Å. , C. G. Löfgren , and M. U. Nylen . 1970. “Tetracycline‐Induced Enamel Defects in the Rat Incisor.” Archives of Oral Biology 15, no. 7: 645–661. 10.1016/0003-9969(70)90133-0. 5272369

[cre270225-bib-0029] Pandya, M. , and T. G. H. Diekwisch . 2021. “Amelogenesis: Transformation of a Protein‐Mineral Matrix Into Tooth Enamel.” Journal of Structural Biology 213, no. 4: 107809. 10.1016/j.jsb.2021.107809. 34748943 PMC8665087

[cre270225-bib-0030] Primosch, R. E. 1980. “Tetracycline Discoloration, Enamel Defects, and Dental Caries in Patients With Cystic Fibrosis.” Oral Surgery, Oral Medicine, Oral Pathology 50, no. 4: 301–308. 10.1016/0030-4220(80)90411-9. 6935580

[cre270225-bib-0031] Radlanski, R. J. , and H. Renz . 2007. “Insular Dentin Formation Pattern in Human Odontogenesis in Relation to the Scalloped Dentino‐Enamel Junction.” Annals of Anatomy ‐ Anatomischer Anzeiger 189, no. 3: 243–250. 10.1016/j.aanat.2006.11.007. 17534031

[cre270225-bib-0032] Ravindra, D. , G. Huang , K. Hallett , D. P. Burgner , A. Gwee , and M. J. Silva . 2023. “Antibiotic Exposure and Dental Health: A Systematic Review.” Pediatrics 152, no. 1: e2023061350. 10.1542/peds.2023-061350. 37264510

[cre270225-bib-0033] Reagan‐Shaw, S. , M. Nihal , and N. Ahmad . 2008. “Dose Translation From Animal to Human Studies Revisited.” FASEB Journal 22, no. 3: 659–661. 17942826 10.1096/fj.07-9574LSF

[cre270225-bib-0034] Sabah, T. , and N. Al‐Ghaban . 2023. “Evaluation of the Effects of Amoxicillin on Tooth Development in Rats by Histological and Histomorphometric Study.” Archives of Razi Institute 78, no. 4: 1333–1341. 38226389 10.32592/ARI.2023.78.4.1333 PMC10787917

[cre270225-bib-0035] Sasaki, T. , M. Goldberg , S. Takuma , and P. R. Garant . 1990. “Cell Biology of Tooth Enamel Formation. Functional Electron Microscopic Monographs.” Monographs in Oral Science 14: 1–199. 2407942

[cre270225-bib-0036] Schmalfuss, A. J. , A. Sehic , and I. J. Brusevold . 2022. “Effects of Antibiotics on the Developing Enamel in Neonatal Mice.” European Archives of Paediatric Dentistry 23, no. 1: 159–168. 10.1007/s40368-021-00677-4. 34716571 PMC8926962

[cre270225-bib-0037] Seow, W. 2014. “Developmental Defects of Enamel and Dentine: Challenges for Basic Science Research and Clinical Management.” Australian Dental Journal 59: 143–154. 24164394 10.1111/adj.12104

[cre270225-bib-0038] Shekarchizadeh, H. , M. R. Khami , S. Z. Mohebbi , H. Ekhtiari , and J. I. Virtanen . 2013. “Oral Health of Drug Abusers: A Review of Health Effects and Care.” Iranian Journal of Public Health 42, no. 9: 929–940. 26060654 PMC4453891

[cre270225-bib-0039] de Souza, J. F. , M. Gramasco , F. Jeremias , et al. 2016. “Amoxicillin Diminishes the Thickness of the Enamel Matrix That Is Deposited During the Secretory Stage in Rats.” International Journal of Paediatric Dentistry 26, no. 3: 199–210. 10.1111/ipd.12184. 26148818

[cre270225-bib-0040] Umamageswari, J. , S. Balasubramanian , K. Krishnakumar , S. Preetha , and K. Vijayarani . 2020. A Simple and Rapid Staining Technique to Confirm Mating in Wistar Rats.

[cre270225-bib-0041] Le Vavasseur, B. , and V. Zeller . 2022. “Antibiotic Therapy for Prosthetic Joint Infections: An Overview.” Antibiotics (Basel, Switzerland) 11, no. 4: 486. 10.3390/antibiotics11040486. 35453237 PMC9025623

[cre270225-bib-0042] Wada, K. , M. Ijbara , N. A. Salim , J. Wada , and T. Iwamoto . 2023. “Three‐Dimensional Microscopic Comparison of Wear Behavior Between Immature and Mature Enamel: An In Vitro Study.” BMC Oral health 23, no. 1: 40. 10.1186/s12903-023-02751-3. 36694188 PMC9875398

[cre270225-bib-0043] Wuollet, E. , S. Laisi , E. Salmela , A. Ess , and S. Alaluusua . 2016. “Molar‐Incisor Hypomineralization and the Association With Childhood Illnesses and Antibiotics in a Group of Finnish Children.” Acta Odontologica Scandinavica 74, no. 5: 416–422. 10.3109/00016357.2016.1172342. 27140829

[cre270225-bib-0044] Yoshizaki, K. , S. Fukumoto , D. D. Bikle , and Y. Oda . 2020. “Transcriptional Regulation of Dental Epithelial Cell Fate.” International Journal of Molecular Sciences 21: 8952. 10.3390/ijms21238952. 33255698 PMC7728066

